# The utility of STOP-BANG questionnaire in the sleep-lab setting

**DOI:** 10.1038/s41598-019-43199-2

**Published:** 2019-04-30

**Authors:** Wojciech Kuczyński, Łukasz Mokros, Aleksandra Stolarz, Piotr Białasiewicz

**Affiliations:** 1 Department of Sleep Medicine and Metabolic Disorders, Medical University of Lodz, Lodz, Poland; 2 Department of Clinical Pharmacology, Medical University of Lodz, Lodz, Poland; 3 Department of Microbiology and Laboratory Medical Immunology, Medical University of Lodz, 92-213 Lodz, Poland

**Keywords:** Diagnosis, Respiratory signs and symptoms

## Abstract

Polysomnography (PSG) is considered the gold standard in obstructive sleep apnea-hypopnea syndrome (OSAS) diagnostics, but its availability is still limited. Thus, it seems useful to assess patients pre-diagnostic risk for OSAS to prioritize the use of this examination. The purpose of this study was to assess positive (PPV) and negative (NPV) predictive values of the STOP BANG questionnaire (SBQ) in patients with presumptive diagnosis of OSAS. From a database of 1,171 (880 men) patients of a university based sleep center, 1,123 (847 men) met eligibility criteria and their SBQ scores were subject to the Bayesian analysis. The analysis of PPV and NPV was conducted at all values of SBQ for all subjects, but also separately for males and females, and for total sleep time (TS) and for sleep in the lateral position (LP). The probability of OSAS (AHI ≥ 5) and at least moderate OSAS (AHI ≥ 15) for TS was 0.766 and 0.516, while for LP the values were 0.432 and 0.289, respectively. Overall, due to low specificity, SBQ had low PPV for TS and LP. Negative test result (SBQ < 3) revealed NPV of 0.620 at AHI < 5 and 0.859 at AHI < 15 for TS, while in LP NPV values were 0.935 at AHI < 5 and 1.0 at AHI < 15, (n = 31), while SBQ < 4 generated NPV of 0.943 in LP (n = 105). SBQ did not change probabilities of OSAS to confirm or rebut diagnosis for TS. However, it is highly probable that SQB can rule out OSAS diagnosis at AHI ≥ 15 for LP.

## Introduction

Obstructive sleep apnea-hypopnea syndrome (OSAS), manifesting with repeated interruptions of ventilation during sleep in the form of apneas or hypopneas, is a prevalent sleep disorder which remains under-diagnosed and, consequently, under-treated. Its reported prevalence differs widely from 2 and 5% to almost 61% and 84% in women and men, respectively^1–3^. Several population-based studies have reported that OSAS escalates the risks of hypertension, vascular diseases, as well as metabolic and endocrine disorders, underscoring the need for a timely diagnosis and treatment^4–7^. One of endotypes recognized among OSAS patients is a positional disease, characterized by disordered breathing, occurring mainly in the supine sleeping position^8,9^. Therefore, diagnosing positional OSAS may have some clinical merit as avoiding sleep in the supine position may be a simple measure to treat this disorder^10,11^.

Polysomnography (PSG) is a diagnostic gold standard, however, not widely available. Therefore it seems useful to assess the individual risk of OSAS, i.e. by dividing patients into low and high risk groups, to prioritize its use. In effect, numerous papers have been published so far on the diagnostic value of single signs and symptoms or their combinations grouped in questionnaires^12–15^. One of them is STOP-BANG questionnaire (SBQ), based on a set of four symptoms and four signs. It was originally developed to stratify post-operative complication risk related to OSAS in a group of adult patients before a major surgery^16,17^. A recent meta-analysis proved that SBQ is a superior tool for detecting mild, moderate, and severe OSAS compared to other questionnaires but the clinical merit was affected by the population on which it was tested^18^. In comparison to a general population, patients referred to a sleep clinic are typically more symptomatic with higher prevalence of OSAS, which may affect the validity of SBQ^18^. Indeed, some investigators reported limited utility of SBQ in this group of patients^19–21^. We have recently reported that a single variable – BMI, can change pre-test probability of OSAS in a population of patients referred to a sleep center due to a presumptive diagnosis of OSAS. Because of high sensitivity of BMI in this group of patients, especially observed in the lateral sleeping position (LP), it was possible to exclude with high probability (98%) moderate or severe OSAS (apnea-hypopnea index, AHI ≥ 15) in LP, identifying a group of patients who could be qualified for positional treatment, even before making a PSG-based diagnosis^22^. The exclusion of at least moderate OSAS in LP based on normal BMI may be of some clinical value, as a PSG-based diagnosis is usually delayed due to limited resources. As BMI is only one of eight clinical variables, used in SBQ, we wondered whether the latter could make a more substantial change regarding the probability of OSAS occurrence, probably restricted not only to the sleep in the lateral position. Thus, we hypothesized that SBQ could have a high negative predictive value in identifying a subgroup of patients with low OSAS probability because of potential high sensitivity. Moreover, as male gender adds one point to SBQ score, we wondered whether the sex could differentiate SBQ performance. To the best of our knowledge, SBQ has not been used yet to assess the probability of OSAS in LP, and affecting a particular sex in a population referred to a sleep center, due to a presumptive diagnosis of OSAS.

## Methods

We performed a retrospective study in the sleep center of the Department of Sleep Medicine and Metabolic Disorders, Medical University of Lodz, Poland. The database included 1,171 patients who underwent polysomnography between 2013 and 2015. All the patients were referred due to a presumptive diagnosis of OSAS, based on typical, not mutually exclusive complaints, including: snoring (78.6%), apneas (72.3%), sleepiness (50.6%) or unrefreshing sleep (32.6%). The SBQ scoring was based on the symptoms and signs that were part of our standard patient’s chart, filled in at the first ambulatory visit. After exclusion, of 48 patients, there were1,123 patients left who were eligible for an analysis (847 males). Exclusion criteria were the following: sleep time duration shorter than 3 hours (n = 36) or absence of at least one of the SBQ items (n = 12). For the analysis in the lateral sleeping position, only 977 patients were eligible, because 146 patients had less than 0.5 h sleep duration in LP, which was arbitrarily considered too low to assess the valid AHI index for this position. All patients gave their informed consent to the sleep study. The Ethics Committee of Medical University of Lodz approved the study protocol (RNN/23/15/KE).

### Polysomnography

Patients were admitted to the sleep center at 21:00 hours (±0.5 hour) and were subjected to general physical examination, which involved measuring the body weight and height for the purpose of BMI calculation. They were ready to fall asleep between 22:00 and 23:00 hours due to approximately 0.5–1.0 hour needed to place sensors and electrodes. A standard nocturnal PSG was performed by recording the following channels: electroencephalography (C4\A1, C3\A2), chin muscles and anterior tibialis electromyography, electrooculography, measurements of oro-nasal air flow (a thermistor gauge), snoring, body position (a gravitational gauge placed on the sternum), respiratory movements of the chest and abdomen (piezoelectric strain gauges), unipolar electrocardiogram and hemoglobin oxygen saturation (SaO2) (Sleep Lab, Jaeger – Viasys, Hoechberg, Germany). Sleep stages were scored according to criteria, based on a 30 s epoch standard^23^. Apnea was attained after reducing air flow to less than 10% of the baseline, for at least 10 seconds. Hypopnea was defined as at least 30% reduction of air flow accompanied by 4% or greater decrease in SaO2 or an arousal^24^. All methods were carried out in accordance with relevant guidelines and regulations; electroencephalography arousals were scored according to American Academy of Sleep Medicine guidelines^25^.

### Study variables

Sleep study evaluation provided variables related to sleep disordered breathing, i.e. a standard AHI calculated for total sleep time (AHI-total) and separately, for sleep in the lateral position (AHI-side). Relevant PSG and clinical variables are presented in Table 1.

**Table 1:** Summary of clinical and sleep study variables.

variable	mean ± SD, or median (IQR)*	males	females	p
M/F [n]	847/276	847	276	
BMI [kg/m^2^]	32.3 ± 6.3, n = 1123	32.3 ± 6.1, n = 847	32.1 ± 6.9, n = 276	0.57
age [years]	52.2 ± 11.9, n = 1123	50.3 ± 11.8, n = 847	57.8 ± 10.1, n = 276	<0.001
sleep duration [h]	5.8 ± 1.1, n = 1123	5.8 ± 1.1, n = 847	5.7 ± 1.1, n = 276	0.23
sleep duration in the lateral position [h]^†^	3.2 ± 1.4, n = 977	3.2 ± 1.4, n = 744	3.0 ± 1.5, n = 233	0.07
sleep duration in the supine position [h]^†^	3.1 ± 1.7, n = 1019	3.1 ± 1.7, n = 767	3.3 ± 1.6, n = 252	0.11
AHI_total_ [n/h]	16.0 (5.8–43.1), n = 1123	19.3(6.8–49.6), n = 847	9.9 (2.7–29.2), n = 276	<0.001
AHI_side_ [n/h]^†^	3.0 (0.3–19.0), n = 977	3.6 (0.4–25.0), n = 744	2.0 (0.2–9.0), n = 233	<0.001
AHI_back_ [n/h]^†^	33.3 (11.0–66.0), n = 1019	42.0 (14.3–68.6), n = 767	16.9 (4.8–39.0), n = 252	<0.001
AHI_total_ distribution [n/h]	n (% of subjects)			
<5	263 (23.4)	166 (19.6)	97(35.1)	<0.001
≥5–5<	280 (24.9)	208 (24.6)	72 (26.1)	<0.001
≥15–30<	185 (16.5)	143 (16.9)	42 (15.2)	<0.001
≥30	395 (35.2)	330 (39.0)	65 (23.6)	<0.001
AHI_side_ distribution [n/h]	n (% of subjects)			
<5	555 (58.8)	403 (54.2)	152 (65.2)	<0.001
≥5–15<	140 (14.3)	102 (13.7)	38 (16.3)	<0.001
≥15–30<	80 (8.2)	66 (8.9)	14 (6.0)	<0.001
≥30	202 (20.7)	173 (23.3)	29 (12.4)	<0.001
STOP BANG parameters	n (% of subjects)			
S**- “Do you SNORE?”**	886 (78.9)	664 (78.4)	222 (80.4)	0.47
**T**- “Do you often feel TIRED during daytime?”	373 (33.2)	288 (34.0)	85 (30.1)	0.33
**O**- “Has anyone OBSERVED you stop breathing during your sleep?”	815 (72.6)	647 (76.4)	168 (60.8)	<0.001
**P** - “Do you have high blood PRESSURE?”	654 (58.2)	483 (57.0)	171 (62.0)	0.15
**B**MI > 35	303 (27.0)	229 (27.0)	74 (26.8)	0.94
**A**ge > 50	704 (62.7)	474 (56.0)	230 (83.3)	<0.001
**N**eck circumference > 40 cm	810 (72.1)	733 (86.5)	77 (27.9)	<0.001
male **G**ender	847 (75.4)	847 (100)	0 (0)	

*Where applicable, i.e. in case of non-normal distribution.

^†^Calculated for the patients with at least 0.5 h sleep duration in the lateral or supine sleep positions.

### STOP BANG questionnaire

SBQ is a tool that combines evaluation of eight items, presumably risk factors for OSAS. It includes a subjective 4-item questionnaire (STOP) and a 4-item clinical assessment (BANG)^16^. SBQ includes four yes/no questions: S - “Do you **S**nore loudly (louder than talking or loud enough to be heard through closed doors)”, T - “Do you often feel **T**ired, fatigued, or sleepy during daytime?”, O - “Has anyone **O**bserved you stop breathing during your sleep?“ and P - “Do you have or are you being treated for high blood **P**ressure?”. The BANG part of the questionnaire consists of four additional demographic queries: body mass index (**BMI**) over 35 kg/m^2^ (B), **A**ge over 50 years, **N**eck circumference over 40 cm, and male **G**ender. For each question, answering “yes” scores 1, a “no” response scores 0, thus the total score ranges from 0 to 8 in men and 0 to 7 in women. We applied the standard cut-off of 3 points as a positive test result. Moreover, we also evaluated all scored levels of SBQ as cut-offs for their ability to change pre-test probability and to find the score with either the highest positive predictive value (PPV) or negative predictive value (NPV) in order to verify OSAS diagnosis. We did it for a standard AHI, calculated for the whole sleep (AHI-total) and separately, for AHI calculated for sleep in the lateral sleeping position (AHI-side), Table 2. As the male sex is one of OSAS risk factors and SBQ items, by definition women scored one point less than men. Therefore, we made a sub-analysis separately for males and females to verify whether this systemic bias influenced the analysis of sensitivity, specificity and predictive values of SBQ in OSAS diagnosis (Tables 3 and 4).

**Table 2:** Sensitivity and specificity with corresponding PPV, NPV, 95%Cl and Youden index for SBQ with different cut- off values from 1 to 8 due to TST > 3.0 hours N = 1135; TST > 3.0, TST(side) > 0.5 hours N = 977.

AHI	SBQ	[N]	sensitivity	95% Cl	specificity	95% Cl	PPV	95% Cl	NPV	95% Cl	Youden in.
TST > 3.0 hours; N = 1135.											
AHI > 5	8	23	3%	(−4–9.2)	100%	100	100%	100	24%	(18.8–29.0)	0,026
	7	149	18%	(11.7–23.7)	93%	(90.1–96.3)	90%	(84.7–94.3)	26%	(20.4–31.2)	0,11
	6	300	47%	(42.6–52.2)	77%	(71.7–83.1)	87%	(84.1–90.5)	31%	(24.8–37.4)	0,249
	5	324	77%	(73.3–79.7)	51%	(42.4–59.2)	84%	(80.7–86.3)	40%	(31.5–48.1)	0,273
	4	213	93%	(90.7–94.3)	23%	(12.3–33.5)	80%	(76.9–82.5)	48%	(35.9–60.9)	0,155
	3	89	99%	(97.7–99.3)	9%	(−2.5 20.5)	78%	(75.2–80.8)	65%	(45.8–84.0)	0,075
	2	35	100%	100	1%	(−11.2–12.8)	77%	(73.9–79.5)	100%	100	0,008
	1	2	100%	100	0%	(−12.0–12.0)	77%	(73.8–79.4)	100%		0
AHI > 15	8	23	3%	(−4.6–11.4)	100%	(98.9–100.0)	87%	(72.3–101.7)	49%	(44.9–53.3)	0,029
	7	149	23%	(16.1–30.3)	93%	(91.3–95.5)	79%	(72.3–85.9)	53%	(49.0–57.6)	0,167
	6	300	55%	(49.5–60.3)	73%	(68.3–77.1)	68%	(63.1–73.3)	60%	(55.4–65.0)	0,276
	5	324	82%	(78.9–85.7)	43%	(36.5–49.1)	61%	(56.2–65.0)	69%	(63.4–75.2)	0,251
	4	213	95%	(93.7–97.1)	18%	(10.4–25.6)	55%	(51.3–59.5)	79%	(70.5–86.7)	0,134
	3	89	99%	(98.2–99.8)	6%	(−2.5–13.7)	53%	(48.7–56.9)	84%	(70.8–96.8)	0,046
	2	35	100%	100	0%	(−7.9–8.7)	52%	(47.7–55.7)	100%	100	0,004
	1	2	100%	100	0%	(−8.4–8.4)	52%	(47.6–55.6)	100%		0
**AHI**	**SBQ**		**sensitivity**	**95% Cl**	**specificity**	**95% Cl**	**PPV**	**95% Cl**	**NPV**	**95% Cl**	**Youden in**.
TST > 3.0 hours and TST_(side)_ > 0.5 hours, N = 997											
AHI > 5	8	19	4%	(−5.8–13.0)	99%	(98.6–100.0)	79%	(58.2–99.6)	58%	(53.4–61.6)	0,028
	7	131	28%	(19.4–35.6)	94%	(91.8–96.0)	77%	(69.7–84.9)	63%	(58.9–67.1)	0,214
	6	251	60%	(53.4–65.6)	73%	(68.7–77.3)	63%	(56.6–68.6)	70%	(65.8–74.8)	0,325
	5	285	84%	(80.1–87.7)	40%	(33.8–46.6)	52%	(46.4–56.8)	77%	(71.0–82.2)	0,241
	4	186	96%	(93.7–97.7)	16%	(8.1–23.3)	46%	(41.4–51.2)	83%	(75.0–90.8)	0,114
	3	74	100%	(98.8–100.2)	5%	(−2.9–13.3)	44%	(39.6–49.2)	94%	(84.5–102.5)	0,048
	2	29	100%	100	0%	(−7.9–8.7)	43%	(38.6–48.0)	100%	100	0,004
	1	2	100%	100	0%	(−8.3–8.3)	43%	(38.5–47.9)			0
AHI > 15	8	19	5%	(−6.4–16.4)	99%	(98.7–99.9)	74%	(50.6–96.8)	72%	(68.6–75.4)	0,042
	7	131	33%	(23.8–42.8)	92%	(89.8–94.0)	63%	(52.9–72.5)	77%	(74.1–80.5)	0,253
	6	251	68%	(61.5–74.7)	70%	(65.8–74.0)	48%	(40.8–55.0)	84%	(81.2–87.6)	0,38
	5	285	90%	(86.4–93.8)	38%	(31.9–43.7)	37%	(31.1–42.9)	90%	(86.8–94.0)	0,279
	4	186	98%	(96.2–99.6)	14%	(7.3–21.1)	32%	(26.2–37.2)	94%	(89.7–98.8)	0,121
	3	74	100%	100	5%	(−2.8–11.8)	30%	(24.5–35.1)	100%	100	0,045
	2	29	100%	100	0%	(−7.1–7.7)	29%	(23.6–34.2)	100%	100	0,003
	1	2	100%	100	0%	(−7.4–7.4)	29%	(23.6–34.2)			0

**Table 3:** Sensitivity and specificity with corresponding PPV, NPV, 95%Cl and Youden index for females with SBQ with different cut- off values from 1 to 7 due to TST > 3.0 hours N = 276; TST > 3.0, TST_(side)_ > 0.5 hours N = 233.

AHI	SBQ	[N]	sensitivity	95% Cl	specificity	95% Cl	PPV	95% Cl	NPV	95% Cl	Youden in.
females; TST > 3.0 hours; N = 276											
AHI > 5	7	9	5%	(−9.2–19.3)	100%	100%	100%	100%	36%	(26.8–45.9)	0,050
	6	19	16%	(2.2–29.1)	100%	100%	100%	100%	39%	(29.4–48.8)	0,156
	5	55	40%	(28.3–51.0)	88%	(80.6–94.6)	86%	(77.4–93.7)	44%	(33.5–54.6)	0,273
	4	69	66%	(57.4–74.5)	65%	(53.2–76.7)	78%	(70.1–85.2)	51%	(38.5–63.2)	0,309
	3	69	89%	(83.9–93.7)	36%	(20.2–52.0)	72%	(65.0–78.9)	64%	(47.7–79.6)	0,249
	2	40	99%	(97.3–100.4)	13%	(−5.1–31.9)	68%	(60.9–74.7)	87%	(68.2–105.1)	0,123
	1	12	100%	100%	3%	(−16.5–22.7)	66%	(58.6–72.5)	100%	100%	0,031
	0	3	100%	100%	0%	(−19.9–19.9)	65%	(57.9–71.8)			0,000
AHI > 15	7	9	8%	(−9.7–26.5)	100%	100%	100%	100%	63%	(56.0–70.6)	0,084
	6	19	20%	(2.6–36.6)	96%	(92.8–98.9)	75%	(56.5–93.5)	65%	(58.0–72.7)	0,155
	5	55	46%	(31.8–59.7)	80%	(73.1–86.6)	59%	(45.3–72.8)	70%	(62.2–77.7)	0,257
	4	69	77%	(67.5–85.8)	59%	(48.9–68.3)	54%	(43.2–64.7)	80%	(71.9–87.7)	0,352
	3	69	93%	(87.3–97.7)	28%	(15.0–40.6)	45%	(35.0–54.6)	85%	(75.4–95.5)	0,203
	2	40	100%	100%	9%	(−5.5–23.3)	41%	(31.7–50.3)	100%	100%	0,089
	1	12	100%	100%	2%	(−13.2–16.7)	39%	(29.9–48.4)	100%	100%	0,018
	0	3	100%	100%	0%	(−15.1–15.1)	39%	(29.5–48.4)			0,000
**AHI**	**SBQ**		**sensitivity**	**95% Cl**	**specificity**	**95% Cl**	**PPV**	**95% Cl**	**NPV**	**95% Cl**	**Youden in**.
females; TST > 3.0 hours and TST_(side)_ > 0.5 hours, N = 233											
AHI > 5	7	4	5%	(−16.3–26.2)	100%	100%	100%	100%	66%	(58.9–73.9)	0,049
	6	30	25%	(5.8–43.6)	91%	(86.0–95.6)	59%	(37.3–80.4)	69%	(61.7–77.0)	0,155
	5	44	48%	(32.5–63.8)	74%	(66.3–82.4)	50%	(34.3–65.7)	73%	(64.7–81.1)	0,225
	4	87	83%	(73.7–91.8)	36%	(22.8–48.3)	41%	(28.8–52.4)	79%	(68.6–90.2)	0,182
	3	45	99%	(96.3–101.2)	14%	(−0.2–29.2)	38%	(27.5–48.7)	96%	(87.1–104.2)	0,132
	2	21	100%	100%	1%	(−14.5–17.1)	35%	(24.7–45.5)	100%	100%	0,013
	1	2	100%	100%	0%	(−15.9–15.9)	35%	(24.4–45.1)			0,000
AHI > 15	7	4	9%	(−19.2–37.8)	100%	100%	100%	100%	83%	(77.6–88.3)	0,093
	6	30	35%	(10.8–59.0)	90%	(85.5–94.5)	44%	(19.0–69.2)	86%	(80.7–91.1)	0,249
	5	44	63%	(44.6–81.0)	73%	(65.8–80.5)	35%	(16.7–52.6)	90%	(84.6–94.7)	0,359
	4	87	88%	(78.2–98.6)	33%	(21.5–44.8)	23%	(9.6–36.4)	93%	(86.2–99.1)	0,215
	3	45	100%	100%	12%	(−1.2–25.4)	20%	(8.4–32.5)	100%	100%	0,121
	2	21	100%	100%	1%	(−13.1–15.2)	19%	(7.0–30.2)	100%	100%	0,011
	1	2	100%	100%	0%	(−14.2–14.2)	18%	(6.9–30.1)		100%	0,000

**Table 4:** Sensitivity and specificity with corresponding PPV, NPV, 95%Cl and Youden index for males with SBQ with different cut- off values from 1 to 8 due to TST > 3.0 hours N = 847; TST > 3.0, TST(side) > 0.5 hours N = 744.

AHI	SBQ	[N]	sensitivity	95% Cl	specificity	95% Cl	PPV	95% Cl	NPV	95% Cl	Youden in.
males; TST > 3.0 hours; N = 847											
AHI > 5	8	29	4%	(−3.2–11.5)	99%	(98.2–100.6)	97%	(89.8–103.3)	20%	(14.0–26.3)	0,035
	7	109	19%	(12.6–26.1)	96%	(93.5–99.3)	96%	(92.2–99.1)	23%	(16.1–29.0)	0,158
	6	209	46%	(40.6–51.6)	80%	(73.3–86.9)	90%	(87.2–93.7)	27%	(19.1–34.1)	0,262
	5	233	74%	(69.7–77.4)	52%	(41.9–62.9)	86%	(83.4–89.4)	33%	(22.7–42.4)	0,260
	4	168	92%	(89.6–93.9)	26%	(12.8–39.0)	84%	(80.7–86.5)	43%	(28.6–58.2)	0,177
	3	83	99%	(98.2–99.7)	5%	(−9.4 20.2)	81%	(78.2–84.1)	56%	(23.8–88.7)	0,044
	2	14	100%	(99.6–100.1)	1%	(−14.6–15.8)	80%	(77.5–83.5)	50%	(−48.0–148.1)	0,005
	1	2	100%	100%	0%	(−15.2–15.2)	80%	(77.4–83.4)			0,000
AHI > 15	8	29	6%	(−3.0–14.5)	99%	(98.7–100.2)	93%	(83.5–102.7)	45%	(40.4–50.5)	0,052
	7	109	23%	(14.7–30.5)	92%	(88.8–94.6)	78%	(69.6–85.4)	48%	(43.1–53.7)	0,143
	6	209	53%	(46.4–58.8)	74%	(68.6–79.0)	72%	(66.2–77.3)	55%	(49.3–61.1)	0,264
	5	233	79%	(75.4–83.6)	45%	(38.0–52.9)	65%	(60.0–69.7)	64%	(56.4–70.9)	0,249
	4	168	95%	(93.2–97.1)	20%	(11.3–29.4)	60%	(55.6–64.7)	77%	(67.3–86.3)	0,155
	3	83	100%	(99.0–100.2)	4%	(−6.2–13.7)	57%	(52.2–61.2)	88%	(70.2–104.8)	0,033
	2	14	100%	100%	1%	(−9.6–10.6)	56%	(51.5–60.5)	100%	100%	0,005
	1	2	100%	100%	0%	(−10.1–10.1)	56%	(51.4–60.3)			0,000
**AHI**	**SBQ**		**sensitivity**	**95% Cl**	**specificity**	**95% Cl**	**PPV**	**95% Cl**	**NPV**	**95% Cl**	**Youden in**.
males; TST > 3.0 hours and TST_(side)_ > 0.5 hours, N = 744											
AHI > 5	8	19	4%	(−6.0–14.8)	99%	(98.0–100.0)	79%	(58.3–99.6)	55%	(50.2–59.9)	0,034
	7	127	33%	(24.1–41.5)	92%	(88.7–94.4)	77%	(68.9–84.5)	62%	(56.7–66.7)	0,244
	6	221	68%	(61.7–73.8)	66%	(60.6–71.9)	63%	(56.7–69.2)	71%	(65.4–76.3)	0,340
	5	241	92%	(89.4–95.3)	27%	(19.0–35.6)	52%	(46.3–57.3)	81%	(73.5–88.2)	0,197
	4	99	99%	(97.7–100.0)	8%	(−1.2–17.5)	48%	(42.3–53.0)	89%	(78.6–99.8)	0,070
	3	29	100%	(99.1–100.3)	2%	(−7.9–11.4)	46%	(40.9–51.5)	88%	(63.0–112.0)	0,014
	2	8	100%	100%	0%	(−9.8–9.8)	46%	(40.5–51.1)			0,000
AHI > 15	8	19	6%	(−6.4–18.2)	99%	(98.1–99.9)	74%	(50.6–96.8)	69%	(64.9–73.0)	0,049
	7	127	38%	(27.6–47.7)	89%	(86.0–91.8)	62%	(51.6–71.7)	75%	(71.1–79.1)	0,266
	6	221	74%	(67.6–80.5)	62%	(57.0–67.7)	48%	(40.9–55.6)	84%	(79.5–87.6)	0,364
	5	241	95%	(92.1–97.8)	25%	(17.0–32.1)	37%	(31.0–43.6)	91%	(86.2–96.2)	0,195
	4	99	100%	(98.8–100.4)	7%	(−1.3–15.5)	34%	(27.7–39.7)	97%	(92.0–102.6)	0,067
	3	29	100%	100%	2%	(−7.1–10.2)	32%	(26.5–38.4)	100%	100%	0,016
	2	8	100%	100%	0%	(−8.7–8.7)	32%	(26.2–38.0)			0,000

### Statistics

The data were analyzed with Statistica 12 (TIBCO Software Inc, Palo Alto, California, United States) with a medical pack. Data distribution was tested with the Shapiro-Wilk test. The predictive values of SBQ score for AHI ≥ 5 and ≥15 events/h were calculated separately for total sleep (TS) and sleep in the lateral position (LP). Moreover, the same analysis was conducted separately for males and females. The ratios with 95% confidence intervals of sensitivity, specificity, PPV, and NPV were calculated by creating 2 × 2 contingency tables; chi square test with Yates correction were used to compare the corresponding values, calculated for TS and LP. A value of P < 0.05 was considered significant.

### Compliance with Ethical Standards

The study was founded by an institutional grant of the Medical University of Lodz 503/0-079-06/503-01. An informed consent was obtained from all individual participants included in the study. The Ethics Committee of Medical University of Lodz approved of the study protocol (RNN/23/15/KE). All methods were carried out in accordance with relevant guidelines and regulations; electroencephalography arousals were scored according to American Academy of Sleep Medicine guidelines.

## Results

Overall, the prevalence of OSAS in patients referred to a sleep center, defined as AHI ≥ 5, accompanied by typical symptoms, was high - 76.6%, while AHI ≥ 15, compatible with at least moderate OSAS diagnosis was observed in 51.6% of subjects. Substantially less patients revealed AHI ≥ 5 in LP, namely 43.2% and only 28.9% of the cohort presented with AHI ≥ 15 in this sleeping position. Detailed clinical and sleep related variables, also in relation to gender, are presented in Table 1.

### Total sleep time analysis

The standard SBQ cut-off of 3 was very sensitive (0.969) but not specific (0.167); therefore, there was a small change in the probability of OSAS diagnosis – PPV for SBQ ≥ 3 was 0.792, while NPV reached 0.620, which was a substantial change from pre-test 0.234 (p < 0.001), but too low to definitely exclude diagnosis. Moreover, only 71 subjects out of 1,123 scored less than 3, which further compromised clinical usefulness of SBQ at this level. Only 29 patients scored 8 and one had AHI < 5 (a false positive); thus, at this level, SBQ was 0.996 specific with PPV of 0.966. The highest Youden index was calculated for SBQ ≥ 5, namely 0.319 which yielded 0.863 and 0.374 PPV and NPV, respectively. Similar results were obtained for at least moderate OSAS (AHI ≥ 15). Three or more points score revealed very high sensitivity (0.983) with low specificity (0.112), which yielded PPV of 0.542 and NPV of 0.859, a substantial change in the probability from 0.484, but still too low to rebut the diagnosis of at least moderate OSAS (p < 0.001). The highest PPV of 0.931 was calculated for SBQ of 8 (n = 29), while the highest Youden index of 0.294 – for SBQ ≥ 5. Results of the Bayesian analysis at different SBQ levels vs. OSAS severity for TS are summarized in Table 2.

### Lateral sleeping position time analysis

In the group of 977 patients, eligible for an analysis in the lateral sleeping position, 422 revealed AHI ≥ 5 which yielded a pre-test probability of 0.432. Similarly, to TS analysis, at the standard cut-off of 3, SBQ was sensitive (0.995) but not specific (0.052) for OSAS diagnosis at AHI ≥ 5, thus in the same manner as for TS, it affected probabilities: PPV of 0.44 was not different from the pre-test probability, while NPV reached 0.935, a substantial rise from pre-test 0.568 (p < 0.001). Nevertheless, akin to TS analysis, the significance of this finding was diminished by the fact that only 31 out of 977 analyzed patients scored less than 3. Up from SBQ score of 3, PPV rose with each level to reach the highest value of 0.79 for SBQ of 8. So, unlike for TS, SBQ did not reach high specificity and, as a result, high PPV; only 19 patients scored 8 but 4 of them were false positive, i.e. they revealed AHI < 5 in this sleeping position. The highest Youden index was observed at SBQ ≥ 6 (0.325) with PPV and NPV of 0.626 and 0.703, respectively. Pre-test probability of not having at least moderate OSAS (AHI ≥ 15) in LP was 0.711 (i.e. 1 – prevalence). All 31 patients that scored less than 3 were true negatives, which yielded NPV of 1.0 (p < 0.001). Interestingly, 105 subjects scored less than 4 (which is about 11% out of 977) and only 6 were false negatives, which contributed to NPV of 0.943 (p < 0.001). Similarly to AHI ≥ 5 in LT, the highest Youden index was calculated at SBQ ≥ 6 with PPV and NPV of 0.479 and 0.844, respectively and PPV at SBQ = 8 was below 0.8. The results of Bayesian analysis at different SBQ levels vs. OSAS severity for sleep in LT are summarized in Table 2.

### Total sleep time analysis in relation to gender

As it could have been expected, the prevalence of OSAS (AHI ≥ 5) and at least moderate OSAS (AHI ≥ 15), was lower in females. It reached 64.9 and 38.8% (n = 276) vs. 80.4 and 55.8% (n = 847) in males, respectively, p < 0.001. SBQ in females at the level ≥ 2, which corresponds to ≥3 in men, was of similar high sensitivity but low specificity (Tables 3 and 4). Interestingly, 28 women (10.1%) scored ≥6 and they were all true positives for AHI ≥ 5, which yielded PPV of 1.0 and was similar to the value of 0.957, calculated for men at SBQ ≥ 7. Conversely, 15 women (5.4%) scored less than 2 and they were true negatives for AHI ≥ 15, allowing for NPV of 1.0, while the same value of NPV was obtained only for 2 men that scored 1 (0.2%).

### Lateral sleeping position time analysis in relation to sex

Akin to the TS analysis, the prevalence of OSAS (AHI ≥ 5) and at least moderate OSAS (AHI ≥ 15) in LP was lower in females. It reached 34.8 and 18.5% (n = 233) vs. 45.8 and 32.1% (n = 744) in men, respectively, p = 0.004 and <0.001. SBQ < 3 in females for AHI ≥ 5 revealed high NPV of 0.957, i.e. only 1 patient out of 23 that scored less than 3, had AHI ≥ 5 (a false negative); while the NPV for AHI ≥ 15 was 1.0 at this level of SBQ – all 23 patients were true negatives, out of 233 females feasible for the analysis. In males, at SBQ ≥ 3 for AHI ≥ 5, only 8 patients scored less and one was a false negative, which yielded NPV of 0.875; for AHI ≥ 15 at this level, NPV was 1.0 but the value of this finding was diminished by the fact that only 8 males out of 744 (1.1%) scored less than 3 in SBQ. PPV in males never exceeded 0.8, while in females for SBQ of 7, it was 1.0; however, only 4 women scored that high. All detailed results concerning the analysis related to gender are shown in Tables 3 and 4.

## Discussion

Originally, SBQ was conceived as a screening tool for surgical patients to identify those with a substantial risk of complications, probably related to OSAS, in order to provide adequate post-surgery care^16,26^. The aim of our study was to evaluate SBQ as a tool which would enable to stratify patients into groups with high and low risk for OSAS. Consequently, we wondered whether this screening tool can be of any value when applied to a different population, i.e. the one that has already been screened by typical signs and symptoms and in consequence, with relatively high OSAS prevalence, and in effect, pre-test probability.

The pre-test probability of OSAS, as defined by AHI ≥ 5 plus typical symptoms (e.g. daily somnolence, unrefreshing sleep), was substantially higher in our cohort (ca 77%) than it was reported for a general population - from 5 to 60%, depending on diagnostic criteria and the studied population^1–3^. So, it seems quite improbable that SBQ would have such a high specificity to yield positive predictive value close to 1.0, thereby confirming diagnosis without a need for PSG evaluation. Conversely, it can be expected that as a high sensitivity screening tool, it can yield high negative predictive value, identifying a subgroup of patients with very low OSAS probability that do not urgently need a PSG examination. And indeed, our analysis showed that SBQ can rule in or rule out diagnosis but only at extreme values, which makes its clinical usefulness doubtful, due to a low number of subject scoring so low or high.

Our data are consistent with those obtained in a recently published meta-analysis, which reported sensitivity of 0.95 at SBQ ≥ 3 for AHI > 5. We observed sensitivity of 0.97, but due to low specificity, resulting PPV was only 0.78, which did not change the pre-test probability of 0.76^18^. Another study, reporting relatively high sensitivity at the cost of low specificity, was conducted on a group of patients with atrial fibrillation^27^. A very similar study regarding the type of population and the number of subjects was conducted by Farney *et al*.^28^. However, their results are different from ours, since more patients who underwent the SBQ, scored less than 3 (222 out of 1,426, i.e. 15.6%). However, this discrepancy may be related to a higher percentage of women in the cohort, namely 42%, who on average, score at least 1 point less due to the SBQ construct. This was supported by our data, e.g. for TS analysis, 55 females (19.9%) scored less than 3 vs. only 16 males (1.9%). Moreover, authors included all patients that underwent PSG, not only those suspected of OSAS. However, similarly to our results, NPV of SBQ < 3 was affected by a large number of false negatives – 134 (60%) at the AHI level of 5, and 63 (28%) at the AHI level of 15. Another study comparing the value of different questionnaires vs. polygraphy/polysomnography yielded similar results of SBQ sensitivity, specificity and related predictive values taking into account higher pre-test probability of OSAS (90%) and at least moderate OSAS (69%)^29^. In a recently published meta-analysis that summed up 11 studies, conducted on sleep clinic populations, the pooled sensitivity and specificity were 0.90 and 0.49, and 0.94 and 0.34 for diagnosis of OSAS or at least moderate OSAS, respectively^20^. These data differ substantially from those originally published by Chun *et al*.^16^, who reported much higher specificity (60 and 53%, at AHI ≥ 5 and AHI ≥ 15, respectively) in a population of in-hospital, pre-surgical patients. These discrepancies can be plausibly explained by differences in the investigated populations.

We also included AHI level of at least 15 in our analysis. It was done on purpose, as this level is generally accepted for commencement of CPAP treatment^30,31^. The majority of patients scored ≥3 and the change in probability in favor of diagnosis, or conversely, against it, was too low to change the decisions regarding the need for PSG examination. According to rules of the Bayesian analysis, any diagnostic test has the highest chance to influence the probability of diagnosis both ways if the pre-test probability is around 0.5. In our study group, this requirement was met for the analysis of OSAS in LP, where NPV was above 0.9 and 1.0 for AHI ≥ 5 and AHI ≥ 15, respectively at SBQ < 3. But in our previous report, similar results were revealed for normal BMI; BMI had high sensitivity >90% for AHI > 5 and AHI > 15 in LP and we concluded that normal BMI alone can be a useful diagnostic tool to exclude patients with AHI ≥ 15 in the lateral sleeping position^32^. As BMI is a part of SBQ, it was reasonable to expect that adding other clinical variables could make this tool more powerful at changing the disease probability. This was also a rationale for adding BANG part to STOP by Chung *et al*., who expected to increase sensitivity and NPV^16^. Nevertheless, in the total sleep analysis of our cohort, NPV at SBQ ≥ 3 did not reach probability >0.9 for AHI ≥ 5 or AHI ≥ 15, and only a small fraction of patients scored less than 3, which makes clinical usefulness of SBQ doubtful in this population. It fared better in the lateral sleeping position; NPV of SBQ ≥ 3 for AHI ≥ 15 in LP reached 1.0, but akin to TS analysis, only 31 (3%) patients scored less. When the level of SBQ was raised to ≥4, 105 patients (11%) scored 3 or less and at this level, NPV was 0.943. To compare, in our previous study, normal BMI alone (≤25 kg/m^2^) yielded similar NPV, i.e. 0.975 when applied to the same population. So it seems that in order to exclude OSAS diagnosis in LP, normal BMI is similar or even better than the combination of symptoms and signs in SBQ. One explanation for the absence of difference in favor of SBQ versus sole BMI may be the fact, that BMI level that scored positive in SBQ was arbitrarily set at the level of at least 35 kg/m^2^, substantially higher than normal BMI level used in our previous study. Another issue with the SBQ construct is the sex-related bias which moves women with similar symptoms, demography and history of hypertension one point down. This definitely influences the Bayesian analysis and, in consequence, must affect PPV and NPV at a given cut-off point. For example, high risk women would score maximum 7 which impedes specificity and related PPV of the SBQ score <8. Conversely, at the level <3, a higher SBQ score in women could have contributed to, lower sensitivity and, and in effect, lower, NPV. And indeed, what seems interesting, in our gender sub-analysis, higher percentage of women revealed extremes of SBQ score, i.e. ≥6 or <3, and at these levels, PPV or NPV usually reached a value of 1.0, which was not noted in men. Therefore, SBQ may be a more reliable tool to exclude OSAS diagnosis in females, but this finding needs to be confirmed in a prospective study.

Our analysis for TS was similar to results of other studies, conducted on similar populations of patients, referred to a sleep lab with a presumptive OSAS diagnosis. Chung *et al*. suggested that SBQ does not provide sufficient predictive values for individual clinical decision-making, regarding the need or urgency for PSG and therapy^32^. Overall, despite high sensitivity, low specificity was responsible for low PPV. Conversely, NPV was quite high but at the cut-off level of 3 it was compromised by a low number of patients scoring less in this population. At the SBQ level of 4 or more, NPV was similar to normal BMI and at this level, the number of referred patients, scoring less than 4 or with normal BMI, was comparable, namely ca 10%. Alternatively, a recent meta-analysis proved that SBQ is a superior tool for detecting mild, moderate, and severe OSAS in comparison to other questionnaires, but overall, its clinical value seems limited, especially in the sleep lab setting^18^.

### Advantages and limitations of this study

SBQ as a diagnostic tool is not sensitive or specific enough to yield high NPV or PPV values in a population with high prevalence of OSAS, due to a substantial number of false negatives and false positives. This quite accurately repeats the results of previous studies conducted on similar populations of patients, referred to a sleep lab^28,29^. Nevertheless, we expanded the study by providing the analysis of OSAS in the lateral sleeping position and sex and obtained interesting results concerning changes in OSAS probabilities.

One of weaknesses of the present study is a selection bias as our results apply to a preselected group, i.e. Caucasian patients with high pre-test probability of disease, who are not really representative for the general adult population. However, this was our intention to test the utility of SBQ on the population, referred to a sleep lab with a presumptive diagnosis of OSAS. Another one is a retrospective design of the study; our results need to be repeated in a prospective study.

In conclusion, it seems that patients referred to a sleep lab are pre-screened by the signs and symptoms which render this population with high OSAS prevalence. SBQ applied to this selected population is not specific enough to significantly change pre-test probability of disease at the most frequent SBQ score levels from 3 to 5. Conversely, it has some value, as patients scoring less than 4, and especially women demonstrate very low probability of at least moderate OSAS in the lateral sleeping position. Positional treatment, although still a subject of an ongoing debate regarding the method, feasibility criteria, long-term efficacy and compliance, can be an option in patients with this OSAS endotype. In clinical practice, it means that a positional treatment can be safely tried in this selected group of low SBQ score while awaiting PSG assessment^10,11^.

## Data Availability

The Excel data, used to support the findings of this study, may be released upon application to the Medical University of Lodz; the contact person - wojciech.kuczynski@umed.lodz.pl at the Department of Sleep Medicine and Metabolic Disorders.
